# Task-Related c-Fos Expression in the Posterior Parietal Cortex During the “Rubber Tail Task” Is Diminished in Ca^2+^-Dependent Activator Protein for Secretion 2 (*Caps2*)-Knockout Mice

**DOI:** 10.3389/fnbeh.2021.680206

**Published:** 2021-06-10

**Authors:** Makoto Wada, Kouji Takano, Masakazu Ide, Yoshitake Sano, Yo Shinoda, Teiichi Furuichi, Kenji Kansaku

**Affiliations:** ^1^Developmental Disorders Section, Department of Rehabilitation for Brain Functions, Research Institute of National Rehabilitation Center for Persons with Disabilities, Tokorozawa, Japan; ^2^Systems Neuroscience Section, Department of Rehabilitation for Brain Functions, Research Institute of National Rehabilitation Center for Persons with Disabilities, Tokorozawa, Japan; ^3^Department of Applied Biological Science, Tokyo University of Science, Noda, Japan; ^4^Department of Environmental Health, School of Pharmacy, Tokyo University of Pharmacy and Life Sciences, Hachioji, Japan; ^5^Department of Physiology, School of Medicine, Dokkyo Medical University, Mibu, Japan; ^6^Center for Neuroscience and Biomedical Engineering, The University of Electro-Communications, Chofu, Japan

**Keywords:** body image, autism model mouse, body ownership illusion, c-Fos imaging, autism

## Abstract

Rubber hand illusion (RHI), a kind of body ownership illusion, is sometimes atypical in individuals with autism spectrum disorder; however, the brain regions associated with the illusion are still unclear. We previously reported that mice responded as if their own tails were being touched when rubber tails were grasped following synchronous stroking to rubber tails and their tails (a “rubber tail illusion”, RTI), which is a task based on the human RHI; furthermore, we reported that the RTI response was diminished in *Ca^2+^-dependent activator protein for secretion 2-*knockout (*Caps2*-KO) mice that exhibit autistic-like phenotypes. Importance of the posterior parietal cortex in the formation of illusory perception has previously been reported in human imaging studies. However, the local neural circuits and cell properties associated with this process are not clear. Therefore, we aimed to elucidate the neural basis of the RTI response and its impairment by investigating the c-Fos expression in both wild-type (WT) and *Caps2*-KO mice during the task since the c-Fos expression occurred soon after the neural activation. Immediately following the delivery of the synchronous stroking to both rubber tails and actual tails, the mice were perfused. Subsequently, whole brains were cryo-sectioned, and each section was immunostained with anti-c-Fos antibody; finally, c-Fos positive cell densities among the groups were compared. The c-Fos expression in the posterior parietal cortex was significantly lower in the *Caps2*-KO mice than in the WT mice. Additionally, we compared the c-Fos expression in the WT mice between synchronous and asynchronous conditions and found that the c-Fos-positive cell densities were significantly higher in the claustrum and primary somatosensory cortex of the WT mice exposed to the synchronous condition than those exposed to the asynchronous condition. Hence, the results suggest that decreased c-Fos expression in the posterior parietal cortex may be related to impaired multisensory integrations in *Caps2*-KO mice.

## Introduction

In the rubber hand illusion (RHI) task, a human participant feels as if a rubber hand becomes one’s own hand when the rubber hand and the participant’s hand are stroked synchronously with two brushes (Botvinick and Cohen, 1998; Armel and Ramachandran, 2003; Tsakiris and Haggard, 2005). In the task, visual (i.e., stroking of the rubber hand) and tactile stimuli (i.e., stroking brush of the unseen participant’s own hand) are simultaneously delivered, and perception of tactile stimuli by the brush gradually moves to the rubber hand by the integration of visual and tactile stimuli; illusory body ownership of the rubber hand subsequently occurs.

This kind of illusory body ownership is sometimes atypical in individuals with autism spectrum disorder (ASD). For example, Paton et al. (2012) reported that participants with ASD showed lesser proprioceptive drifts, which are used as a major indicator for the human RHI task, than did the controls. Furthermore, Cascio et al. (2012) reported that children with ASD initially showed lesser proprioceptive drifts than did the controls; however, the former group showed the effects of the illusion after 6 min. They discussed that the delayed occurrence of the RHI may have resulted from atypical multisensory temporal integration.

Several neuroimaging studies with neurotypical human participants have suggested that multisensory temporal integration in the posterior parietal cortex (PPC) is critical for the occurrence of the RHI and that the activation of the ventral premotor cortex is related to the illusory body ownership of the rubber hand (Ehrsson et al., 2004, 2005). Modulation of the fronto-parietal connections during the RHI was examined by transcranial magnetic stimulation (TMS) (Karabanov et al., 2017). In addition, a human electrophysiological study using intracranial electrodes has now suggested that increased high-γ (70–200 Hz) activity in the premotor cortex and PPC is related to the illusory body ownership of the rubber hand (Guterstam et al., 2019). These human studies have suggested the importance of the PPC in the formation of illusory perception of the rubber hand at the premotor regions. To investigate the neural basis of the body ownership further, in terms of local neural circuits, cell properties, etc., it becomes necessary to perform invasive measurements in animal models.

Several studies have suggested that the RHI also occurs in macaque monkey (Shokur et al., 2013; Fang et al., 2019). Shokur et al. (2013) have suggested that synchronous visual and tactile stimuli to an avatar’s and a monkey’s hand caused neural activation in the somatosensory cortex when the avatar’s hand is “touched” thorough a virtual reality system. Moreover, Fang et al. (2019) have developed primate version of the RHI using a video-based system. We previously found that even mice show RHI-like response (a “rubber tail illusion”, RTI) (Wada et al., 2016). In the experiment, synchronous stroking of an artificial tail (rubber tail) and the real tail of a mouse was performed using two small brushes (a “rubber tail task” in mice). After >1 min of synchronous stroking, the mice responded (e.g., orienting or retracting the head) as if their own tails were being touched when the rubber tails were grasped. Using the rubber tail task, we also found that the response was weakened in the *Ca^2+^-dependent activator protein for secretion 2-*knockout (*Caps2*-KO) mice (Wada et al., 2019), which are known as ASD model mice (Sadakata et al., 2007a,b; Shinoda et al., 2011). This result is consistent with those of other studies, that is, the occurrence of the RHI is atypical in individuals with ASD or higher autistic traits (Cascio et al., 2012; Paton et al., 2012; Palmer et al., 2013; Ide and Wada, 2017). To clarify the neural basis of the atypical response, we attempted to investigate neural circuits related to the RTI phenomenon.

c-Fos, which encodes a 62 kDa protein, is found as a proto-oncogene, and it responds immediately to extracellular signals (immediate early gene) (Sheng and Greenberg, 1990). In neuronal cells, the expression of c-Fos is induced by an inflow of calcium ions that is accompanied with action potentials, and it is used as one of the activity dependent markers (Morgan et al., 1987; Guzowski et al., 1999). By conducting functional histological studies with immediate-early genes (e.g., c-fos, arc) that are expressed immediately following neural activations (Hirokawa et al., 2008; Wada et al., 2010), we can detect task-related regions at whole section levels. Moreover, after such screening tests, we can identify cell properties and related local neural circuits using double staining.

The previous behavioral study suggests that a large difference in response rate of the synchronous stroking condition was observed between in *Caps2*-KO and wild-type (WT) mice in the rubber tail task (Wada et al., 2019). Therefore, in this study, we investigated c-Fos expression in *Caps2*-KO and WT mice following synchronous stroking, which would cause the RTI response in the WT mice, to investigate the brain regions that are related to the weakened responses in the KO mice. In addition, we also compared the c-Fos expression between WT mice exposed to synchronous and asynchronous stroking to investigate the brain regions that would be related to the RTI response.

## Materials and Methods

### Subjects

Wild-type mice (*n* = 12) and *Caps2*-KO mice (*n* = 8) were used in this study. The mice were derived from our previous study (Wada et al., 2016, 2019). All the WT mice were C57BL/6NCrj (Charles River Laboratories Japan Inc., Yokohama, Japan). All the *Caps2*-KO mice were derived from Tokyo University of Science. Details regarding the production and phenotypes of the KO mice were reported previously, and the mice were characterized based on their autistic behaviors (Sadakata et al., 2007a,b; Shinoda et al., 2011). All the mice in the present study experienced the rubber tail task, and the behavioral results were previously reported (Wada et al., 2016, 2019). Thus, exactly same mice that previously behavioral tested were used in the study. The mice were provided *ad libitum* access to water and food, and were housed in groups of two to four littermates in a room with a 12 h light/dark cycle. The experiment was conducted during the light cycle. Prior to the experiment, the mice had no history of drug administration or surgery, and had experienced the rubber tail tasks that were reported previously (Wada et al., 2016, 2019). The experiments were approved by the institutional committee for animal experimentation (Research Institute of the National Rehabilitation Center for Persons with Disabilities).

### Apparatus

The mice were trained in a stainless-steel tube that was designed for the rubber-tail task (O’Hara, Tokyo, Japan). One side of the tube was open, and the other side of the tube was connected to a transparent plastic cone. A rubber tail was placed on either the right or the left side of the cone. In the control experiment in the previous study (Wada et al., 2016), when the rubber tail was occluded by an opaque plastic plate, the response measured as head movements significantly decreased, suggesting that the mice can see the rubber tail from the cone. Thus, visuotactile integration seems to be important for the RTI phenomenon. The mice were not fixed during the behavioral tasks. The apparatus was essentially identical to those used in our previous study (Wada et al., 2016, 2019).

### Visuotactile Stimulation Before Perfusion (Synchronous or Asynchronous Condition)

**FIGURE 1:**
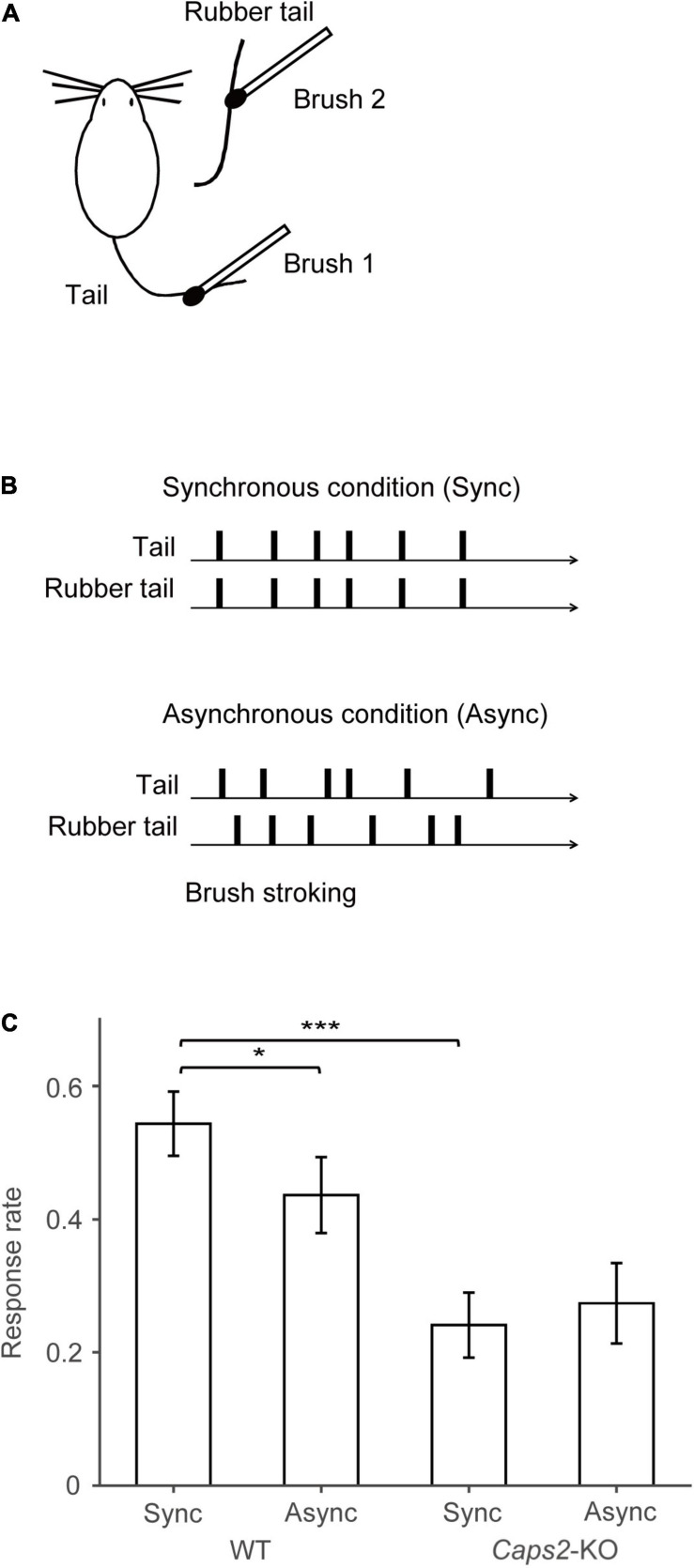
Behavioral task. **(A)** Task set-up: Synchronous or asynchronous stroking of real tails of mice and the rubber tails was performed by using two small brushes. **(B)** Behavioral task: Under the synchronous condition, synchronous stroking of an actual tail and a rubber tail was performed by using two small brushes. In contrast, under the asynchronous condition, mice received asynchronous stroking. Details of the apparatus are essentially identical to those used in our previous study (Wada et al., 2016, 2019). **(C)** Result of the rubber tail task: As reported previously (Wada et al., 2019), in the rubber tail task, the mice were received to 10-min daily tests under two conditions: (1) synchronous stroking of the real tail and a rubber tail (synchronous condition, Sync), and (2) asynchronous stroking of both tails (asynchronous condition, Async). After the tails were stroked for approximately 1 min, an experimenter who was blinded to experimental conditions grasped the rubber tail. The behavioral test consisting 8–10 trials/condition was conducted more than 10 days. Movements of the mice were evaluated by technical staffs who were blinded to experimental conditions using recorded movies. If the mouse’s head immediately turned toward the rubber tail or retracted into the tube, the response was considered a full response with a full score (1.0). If the response was small or delayed (∼1 s), the response was considered as an intermediate response with a partial score (0.5). Moreover, trials in a case, in which the mouse did not respond, were given a score of zero (0). For calculating the response rate of the mice, the moving averages before and after 10 trials (every 21 trials) of the response were calculated. We subsequently extracted the time point at which the difference between the response rates of the synchronous and asynchronous conditions was maximized for each mouse (minimum *p*-values between conditions, one-sample *t*-test) for the response rate in the rubber tail task. Bar graphs indicate averaged response rates from 12 wild-type (WT) mice and 8 *Caps2*-KO mice that were used in the present experiment. The behavioral data and analysis were derived from the previous studies (Wada et al., 2016, 2019). *p < 0.05, ***p < 0.001.

All mice underwent the rubber tail task, and the results were previously reported (Wada et al., 2016, 2019). Four WT mice were derived from the first study (Wada et al., 2016), while eight WT mice and all KO mice were derived from the latter study (Wada et al., 2019). Since all the mice experienced the rubber tail task, the mice were accustomed to receiving synchronous or asynchronous stroking of their tails and the neighboring rubber tails in the small tube (Figure 1A). More than 3 days following the rubber tail task, in the visuotactile stimulation preceding the perfusion (Figure 1B), the mice were placed in the tube for 20 min, and their tail and the rubber tail were brush stroked. Eight KO mice and six WT mice underwent synchronous stroking of their tails (synchronous condition), and the other six WT mice underwent asynchronous stroking of the tails (asynchronous condition). Unlike in the rubber tail task, neither the rubber tail nor the actual tail was grasped by the experimenter before the perfusion to prevent brain activities that could have resulted from the motor responses related to tail grasping.

### Histology

After the 20-min behavioral test, the mice were immediately anesthetized, administered an overdose of pentobarbital intraperitoneally, and perfused intracardially with 60 ml of saline followed by 30 ml of 4% paraformaldehyde in 0.1 M phosphate buffer (Wako Junyaku, Osaka, Japan). The brains were removed and stored overnight in 4% paraformaldehyde at 4°C overnight. Subsequently, the brains were first transferred to 20% sucrose in 0.1 M phosphate buffer and then to 30% sucrose in 0.1 M phosphate buffer at 4°C until they sank. The brain blocks were mounted in OCT compound (Miles Inc., Elkhart, IN, United States), rapidly frozen in a bath (UT-2000F, Tokyo Rikakikai Co, Ltd, Tokyo, Japan) filled with pentane and hexane (1:1) at −100°C, and then stored at −80°C until dissection. Coronal brain sections (7 μm) were sliced on a freezing microtome (CM3050S, Leica, Nussloch, Germany). The sections were mounted on pre-coated slides (MAS-coated slide, Matsunami Glass Ind., Ltd., Osaka, Japan).

### Immunostaining of c-Fos

For the labeling of cells that expressed c-Fos protein, the avidin-biotin peroxidase (ABC) method was used with a polyclonal rabbit antibody specific to c-Fos (SC-52; Santa Cruz Biotechnology, Santa Cruz, CA, United States). The sections were first treated with 0.3% H_2_O_2_ in methanol for 30 min to destroy endogenous peroxidases and then incubated for 20 min with diluted normal blocking serum in phosphate-buffered saline to minimize nonspecific labeling. Subsequently, the tissue sections were incubated for 30 min at room temperature in anti-c-Fos antibody (SC-52) diluted to 1:500 with Da Vinci Green Diluent (Biocare Medical, LLC, Pacheco, CA, United States). The sections were washed, placed in biotinylated goat anti-rabbit antibody diluted to 1:200 for 30 min (Vectastain, ABC Elite kit, Vector Laboratories, Burlingame, CA, United States), washed again, and then placed for 30 min in avidin–biotin complex diluted to 1:200 (Vectastain, ABC Elite kit). The peroxidase activity was visualized with a coloring solution (Histofine DAB Substrate, NICHIREI Bioscience Inc., Tokyo, Japan). After washing with distilled water, the sections were dehydrated through mixtures of ethanol, methanol, and 2-propanol for four washes, transferred to xylene for four washes, and then covered with coverslips. This immunostaining process was conducted on contract (Genetic lab, Sapporo, Japan). The specificity of the antibody (SC-52) had been confirmed in the previous study (Wada et al., 2010).

### c-Fos Imaging

For quantitative analysis, we calculated the densities of c-Fos-positive cells and compared them among the groups according to the previously reported methods (Wada et al., 2016, 2019). The level from the bregma point of each coronal section was determined by using a mouse brain atlas (Paxinos and Franklin, 2001).

Images of each section were acquired using a slide scanner with a bright-field microscope (×20, Nippon Roper, Tokyo, Japan), adjusted such that 1 pixel was equal to 2.5 μm. Scanning of the images was conducted on contract (Genetic lab). For each mouse, neighboring sections were captured at six levels from bregma (+2, +1, −1, −2, −3, and −4 ± 0.2 mm).

The adjusted image of a whole section was fast Fourier transform (FFT) band-pass filtered with an NIH image-J program (NIH, Bethesda, MD, United States) to eliminate low-frequency drifts (>20 pixels = 50 μm) and high frequency noises (<1 pixel = 2.5 μm). The filtered image was further analyzed with a homemade program that was developed on MATLAB with image processing and statistics toolboxes (Mathworks Inc., Natick, MA, United States). As described previously (Wada et al., 2006, 2010), a density map of c-Fos positive cells was prepared for each section by detecting c-Fos positive cells and by calculating the number of immunostained cells in each compartment sized 100 μm × 100 μm, and the map was resized and normalized to a standard section (150 × 200 pixels, 1 pixel ≒ 50 μm). For each mouse, we created c-fos-positive cell density maps (PCDM) at the six levels. A medial–lateral axis of the PCDMs was aligned according to the side on which the rubber tail was placed. Subsequently, we created matrices by piling the maps of each group.

### Statistical Parametric Mapping of the c-Fos-PCDM

Using the matrices, we carried out a pixel-by-pixel between-group comparison of c-Fos-positive cell densities in each matrix of PCDMs. *T*-test was repeatedly applied to each block, after spatially smoothing each map with a Gaussian filter (SD = 8 pixels ≒ 400 μm). We first calculated if the regions where the WT mice were exposed to the synchronous condition (*n* = 6) showed a greater (or lesser) c-Fos cell density than did the *Caps2*-KO mice exposed to the synchronous condition (*n* = 8). The *p*-value of the *t*-test at each point (*p* < 0.001) was mapped to show areas that would reflect the differences between the groups.

Then, we applied the cluster extent threshold to avoid false-positive activities. In this study, we used a two-dimensional cluster because the slice gap (approximately 1 mm) was sufficiently large to ignore effects of other clusters. Thus, from a formula of the cluster extent threshold for the 3-dimensional clustering (Kawaguchi, 2017), we transformed this formula for 2-dimensional clustering by using Euler characteristic densities two-dimensionally. Then, a cluster size, *S*, for the threshold was calculated as follows by using MATLAB:

(1) $$S=\frac{{\text{FWHM}}^{2}\cdot \log p}{A\cdot T_{i\,n\,v}\,{\left(1-\frac{p}{2},\,n_{1}+n_{2}-2\right)}^{2}\cdot \text{Γ}\,\left(2\right)},$$

Where *p* is the corrected *p*-value for multiple comparisons (*p* = 0.05), FWHM is the full width at half maximum (eight pixel), *n*_1_ and *n*_2_ are sample size of each group, and *A* is the constant number, calculated as follows:

$$A=\frac{4\,\log2}{2\,\pi }$$

We further examined the regions where the WT mice exposed to the synchronous condition (*n* = 6) showed a greater (or lesser) c-Fos cell density than did those exposed to the asynchronous condition (*n* = 6) in the same way.

In addition, we carried out a pixel-by-pixel calculation of the Pearson correlation coefficient between the response rate of the synchronous condition in the rubber tail task and c-Fos-positive cell densities in each matrix of PCDMs, using the matrices of the WT and *Caps2*-KO mice exposed to the synchronous condition (*n* = 14). The correlation coefficients (*r*) were displayed with a pseudo-color map (Supplementary Figure 1), while the results of pixels that could not confirm the normality of the dataset using the Kolmogorov-Smironov test (*p* < 0.05) were omitted (colored with black).

## Results

The mice were derived from the previous studies, and all of them experienced the rubber tail task (Wada et al., 2016, 2019). In the rubber tail task, the mice were received to daily tests under two conditions: (1) synchronous stroking of the real tail and a rubber tail (synchronous condition), and (2) asynchronous stroking of both tails (asynchronous condition). As previously reported, in the *Caps2*-KO mice, a strain of the ASD model mice, the RTI response by the rubber tail task was weak (Wada et al., 2019). Considering the mice that were used the present study, the response rates (0.24 ± 0.049, mean ± sem) of the *Caps2*-KO mice [from the previous study (Wada et al., 2019)] were also significantly smaller than those (0.54 ± 0.048) of the WT mice [from the previous study (Wada et al., 2016, 2019)] in the synchronous condition of the rubber tail task (Figure 1C, *t*_17.0_ = –4.40, *p* = 0.00039, Welch’s *t*-test, 95% *confidence interval* = [–0.45, –0.16], *d* = 1.97), while the difference in the response rates (*Caps2*-KO: 0.27 ± 0.060, WT: 0.44 ± 0.057, respectively) were marginal but not significant in the asynchronous condition (*t*_16.6_ = –1.95, *p* = 0.068, Welch’s *t*-test, 95% *confidence interval* = [–0.34, 0.013], *d* = 0.88). In addition, as previously reported (Wada et al., 2016, 2019), the WT mice showed significantly higher response rates under the synchronous condition than under the asynchronous condition (Figure 1C, *t*_11_ = 2.38, *p* = 0.037, one-sample *t*-test, 95% *confidence interval* = [0.0079, 0.21], *d* = 0.69), while there were no significant differences in the response rates under the conditions (*t*_7_ = 0.55, *p* = 0.60, one-sample *t*-test, 95% *confidence interval* = [–0.17, 0.11], *d* = –0.19) in the *Caps2*-KO mice.

From the behavioral results, a large difference in response rate of the synchronous stroking condition was observed between in *Caps2*-KO and WT mice. Therefore, we investigated c-Fos expression in *Caps2*-KO and WT mice following synchronous stroking. In addition, we also compared the c-Fos expression between WT mice exposed to synchronous and asynchronous stroking. More than 3 days after the rubber tail task, synchronous or asynchronous visuotactile stimulation (Figure 1B) and the perfusion were conducted.

### c-Fos-PCDM

**FIGURE 2:**
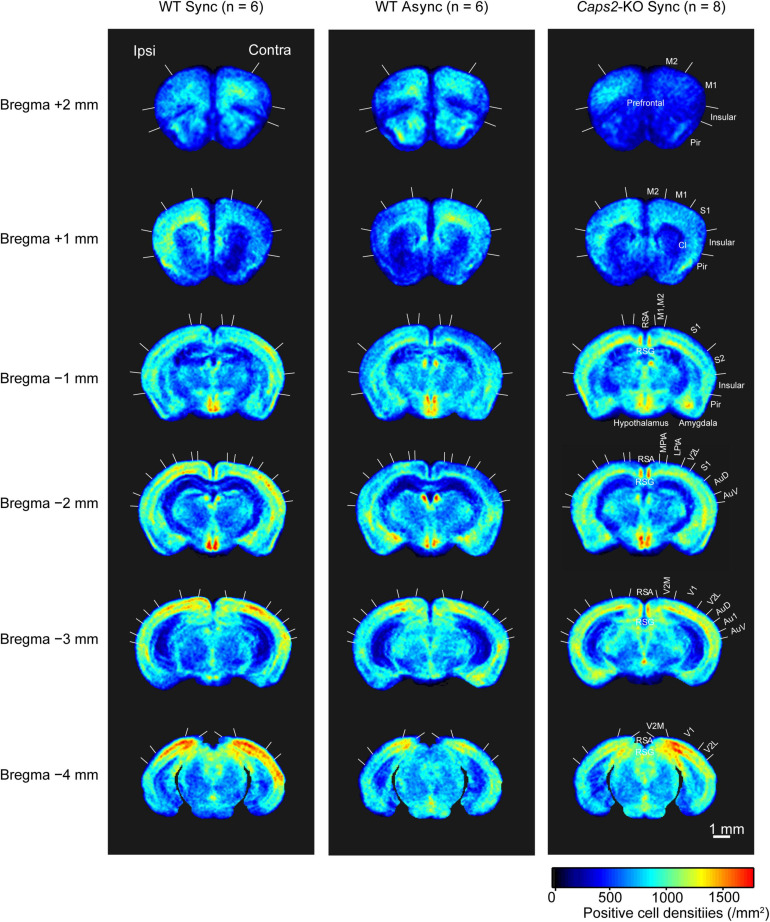
c-Fos-positive cell density maps (PCDM). PCDMs corresponding to six different levels (bregma +2, +1, −1, −2, −3, and −4 mm) are shown in rows for each group (columns: WT with Asynchronous condition, WT with Synchronous condition, *Caps2*-KO with Synchronous condition). Each PCDM map was aligned according to the side of the rubber tail and combined. Boundaries between regions were determined by reference to the brain atlas (Paxinos and Franklin, 2001). M2, secondary motor cortex; M1, primary motor cortex; Pir, Piriform cortex, SI, primary somatosensory cortex; S2, secondary somatosensory cortex; RSA, retrosplenial agranular cortex; RSG, retrosplenial granular cortex; MPtA, medial parietal association cortex; LPtA, lateral parietal association cortex; Au1, primary auditory cortex; AuD, secondary auditory cortex dorsal area; AuV, secondary auditory cortex ventral area; V1, primary visual cortex; V2M, medial secondary visual cortex; V2L, lateral secondary visual cortex. Scale bar: 1 mm.

Figure 2 shows c-Fos-PCDMs at the six levels for the WT mice exposed to the synchronous and asynchronous conditions and the *Caps2*-KO mice exposed to the synchronous condition. A medial–lateral axis of the PCDMs was aligned according to the side on which the rubber tail was placed. In the figure, red color indicates regions where c-Fos-positive cell densities were high (e.g., the primary visual cortex in all three groups).

### Statistical Parametric Mapping of c-Fos-PCDM

**FIGURE 3:**
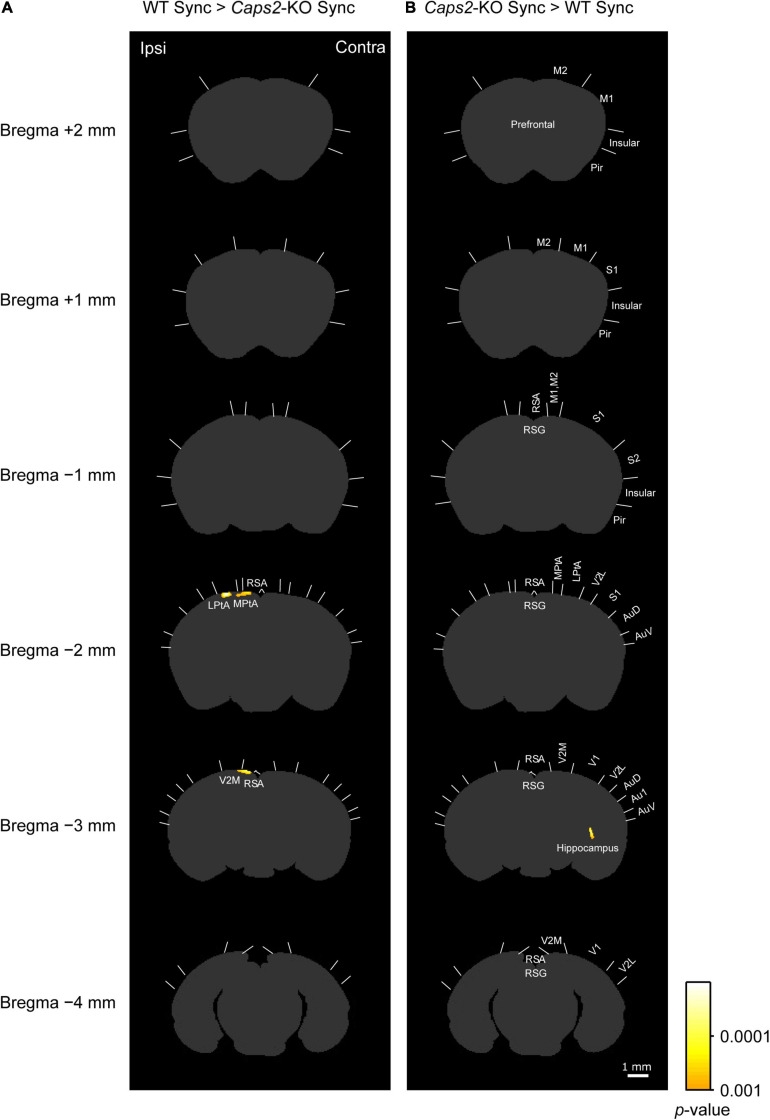
c-Fos imaging (statistical parametric mapping): Comparison between WT and *Caps2*-KO mice exposed to synchronous stroking. **(A)** Areas that showed significantly higher densities of c-Fos-positive cells in the WT mice exposed to the synchronous condition than in the *Caps2*-KO mice exposed to the synchronous condition after correcting for multiple comparisons at the cluster-level threshold (*p* < 0.05). Pixel-by-pixel *t*-tests were applied between two groups. **(B)** Areas that showed significantly higher densities of c-Fos-positive cells densities in the *Caps2*-KO mice exposed to the synchronous condition than in the WT mice exposed to the synchronous condition after the cluster level control. Boundaries between regions were determined by reference to the brain atlas (Paxinos and Franklin, 2001). Scale bar: 1 mm.

**FIGURE 4:**
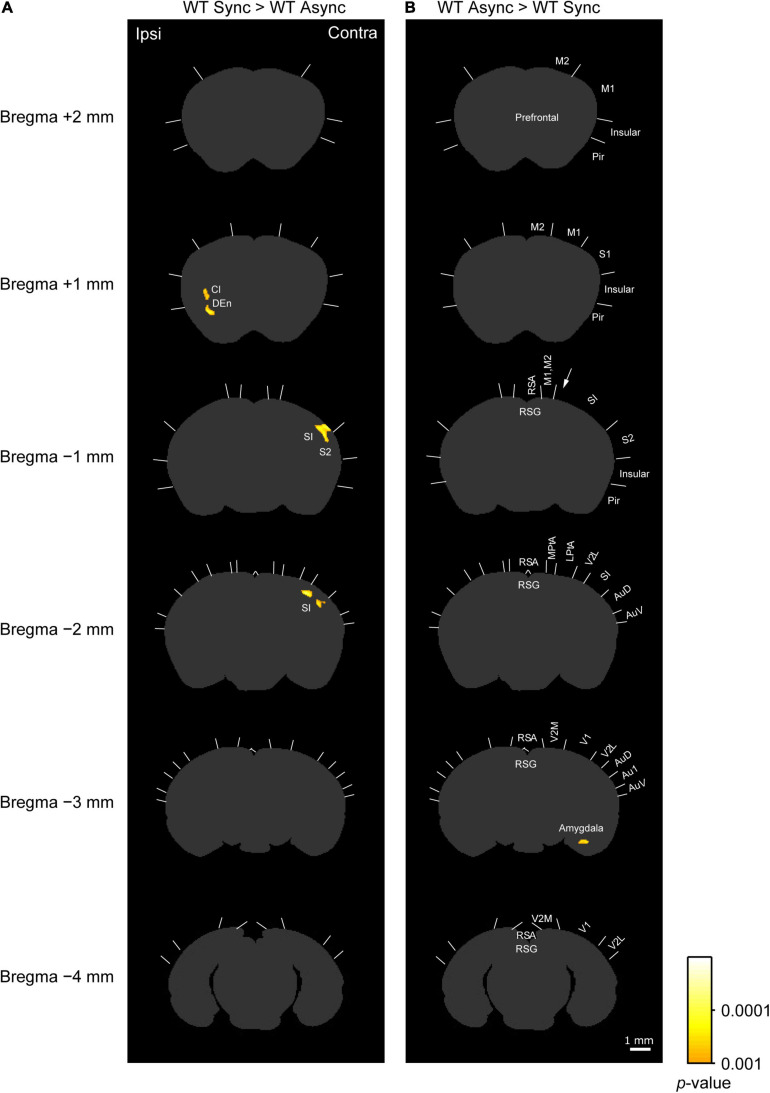
c-Fos imaging (statistical parametric mapping): Comparison between Synchronous and Asynchronous conditions in WT mice. **(A)** Areas that showed significantly higher densities of c-Fos-positive cells in the WT mice exposed to the synchronous condition (WT Sync) than in those exposed to the asynchronous condition (WT Async) after the cluster level control. Pixel-by-pixel t-tests were applied between two groups. **(B)** Areas that showed significantly higher c-Fos-positive cell densities in the WT mice exposed to the asynchronous condition (WT Async) than in those exposed to the synchronous condition (WT Sync) after the cluster level control. Boundaries between regions were determined by reference to the brain atlas (Paxinos and Franklin, 2001). Cl, claustrum; DEn, dorsal endopiriform nucleus. Scale bar: 1 mm.

We compared the densities of c-Fos-positive cells among the groups by using statistical parametric mapping of immuno-positive cell density. Figures 3, 4 show the differences among the groups in detail, because the statistical parametric mapping can detect even small differences in cell densities (Wada et al., 2006).

As shown in a left column of Figure 3 (“WT Sync > *Caps2*-KO Sync”), the WT mice exposed to the synchronous condition showed significantly higher densities of c-Fos-positive cells than did the *Caps2-*KO mice exposed to the synchronous condition in several regions after correcting for multiple comparisons at the cluster-level threshold (*p* < 0.05). Significantly higher densities of c-Fos-positive cells in the ipsilateral posterior parietal association cortex (LPtA, lateral parietal association cortex, bregma −2 mm level) and the ipsilateral retrosplenial agranular cortex (RSA, bregma −2 mm, −3 mm level) were observed in the WT mice than in the *Caps2*-KO mice (Table 1A). Regarding the ipsilateral posterior LPtA cortex (bregma −2 mm level), a small cluster was also observed at the comparison between the synchronous and asynchronous conditions in the WT mice (“WT Sync > WT Async,” *p* < 0.001, uncorrected) in the WT mice, although the cluster size was not reached the cluster-level threshold.

**TABLE 1:** Regions that have significant difference in c-Fos imaging among groups.

Bregma	Region	Minimum *p*	Area (pixel^2^)
**(A) WT Sync > *Caps2*-KO Sync**			
−2 mm	LPtA	0.000011	49
−2 mm	RSA, MPtA	0.00018	56
−3 mm	RSA, V2M	0.000014	68
**(B) *Caps2*-KO Sync > WT Sync**			
−3 mm	Hippocampus	0.000026	31
**(C) WT Sync > WT Async**			
+1 mm	DEn	0.0001	49
+1 mm	Cl	0.00033	41
−1 mm	SI, S2	0.000021	136
−2 mm	SI	0.000048	52
−2 mm	SI	0.0002	36
**(D) WT Async > WT Sync**			
−3 mm	Amygdala	0.00024	34

*Regions that had larger area than the cluster-level threshold (A,B) larger than 28 pixel^2^, (C,D) larger than 25 pixel^2^) are listed. LPtA, lateral parietal association cortex; MPtA, medial parietal association cortex; RSA, retrosplenial agranular cortex; AuD, secondary auditory cortex dorsal area; AuV, secondary auditory cortex ventral area; V2M, medial secondary visual cortex; Cl, Claustrum; DEn, dorsal endopiriform nucleus; SI, primary somatosensory cortex.*

On the contrary, as shown in a right column of Figure 3 (“*Caps2*-KO Sync > WT Sync”), the *Caps2*-KO mice exposed to the synchronous condition showed significantly higher densities of c-Fos-positive cells than did the WT mice exposed to the synchronous condition in the contralateral hippocampus (Table 1B).

As shown in a left column of Figure 4 (“WT Sync > WT Async”), at bregma 1 mm level, densities of c-Fos-positive cells were significantly higher in the ipsilateral claustrum (cl) and dorsal endopiriform nucleus (DEn) in the WT mice exposed to the synchronous condition than those exposed to the asynchronous condition (Table 1C). At bregma −1 mm and −2 mm levels, significantly higher densities of c-Fos-positive cells were observed in the primary somatosensory cortex (SI) in the contralateral side that the rubber tail were presented. According to previous studies (Woolsey, 1967; Krubitzer et al., 2011), the tail region of the SI is located in an anteromedial part (an arrow in Figure 4) adjacent to the motor cortex. There was no significant cluster that reached the cluster-level threshold in the surrounding region.

Furthermore, as shown in a right column of Figure 4 (“WT Async > WT Sync”), densities of c-Fos-positive cells in the WT mice exposed to asynchronous condition were significantly higher than those exposed to the synchronous condition only in the contralateral basal amygdaloid nucleus at bregma −3 mm (Table 1D).

Additionally, with regards to the WT and *Caps2*-KO mice exposed to the synchronous condition, we calculated the Pearson correlation coefficient between the response rate of the synchronous condition in the rubber tail task and c-Fos-positive cell densities in each matrix of PCDMs (Supplementary Figure 1). In addition to the contralateral SI cortex (bregma −1 mm level), ipsilateral LPtA cortex (bregma −2 mm level), higher correlations (*r* > 0.7) were observed at the ipsilateral motor cortex (M1, primary motor cortex; M2, secondary motor cortex; bregma 1 mm level) and contralateral DEn (bregma 2 mm level).

## Discussion

In this study, we found that the c-Fos-positive cell densities in the ipsilateral PPC and adjacent regions under the synchronous conditions were significantly lower in the *Caps2*-KO mice than in the WT mice, and densities of c-Fos-positive cells in the regions were generally higher in the WT mice exposed to the synchronous condition than those exposed to the asynchronous condition, although the difference did not reach a significant level.

In human imaging studies, activations of the PPC, ventral premotor cortex (PMv), lateral occipitotemporal cortex, and extrastriate cortex have been reported to be associated with the RHI (Ehrsson et al., 2004, 2005; Limanowski and Blankenburg, 2015, 2016). Functional connections between the frontal and parietal areas may play an important role in the occurrence of the illusion (Limanowski and Blankenburg, 2015; Arizono et al., 2016; Karabanov et al., 2017). In particular, in humans, the PPC is thought to be important for multisensory integration, which may be involved in the occurrence of RHI (Ehrsson et al., 2005), and attenuation of the illusion was reported after TMS over the PPC (Kammers et al., 2009). In the present study, a significant decrease in the density of c-Fos positive cells in the PPC was observed in the *Caps2*-KO mice that showed impairment of the RTI response (Wada et al., 2019). In addition, the c-Fos-positive cell densities in the motor cortex (M1 and M2) were also correlated with the response rates in the synchronous condition in the rubber tail task, while there was no significant difference in the statistical parametric mapping due to individual variation. The correlation in the higher motor cortex might reflect the illusion related activity like human imaging studies (Ehrsson et al., 2004; Gentile et al., 2013), though it is also possible that the correlation in the M1 simply reflects the motor response of the mice. Based on these results, we speculate that simultaneous visuotactile stimuli by two brushes causes synchronized activities and integration of information in the PPC, and the information would be conveyed to the higher motor cortex. This step is important for the occurrence of the RTI. The decreased c-Fos expression in the PPC under the synchronous condition may be related to impaired multisensory integrations in the *Caps2*-KO mice, although sensory dysfunctions was not reported in this strain (Sadakata et al., 2007a; Shinoda et al., 2011). This might cause weakened RTI responses (Wada et al., 2019). In individuals with ASD, atypical occurrence of the RHI (Cascio et al., 2012; Paton et al., 2012) and atypical multisensory integrations (Stevenson et al., 2014) have been reported. Moreover, previous human imaging studies suggest that multisensory integration in PPC is important for the occurrence of RHI (Ehrsson et al., 2004, 2005; Ehrsson, 2012). The lower activation of PPC by simultaneous visuotactile stimuli in the present study indicates that there is insufficient multisensory integration in this case, and then would relate atypical occurrence of RTI in the ASD model. However, in this study, we examined just one strain (*Caps2*-KO) of ASD model mice, and so evaluations using other ASD models are needed in future studies.

An increase in the density of c-Fos positive cells in the ipsilateral claustrum and adjacent regions were also observed in the WT mice exposed to the synchronous condition. The claustrum is a region of the brain that is a characteristic feature of mammals and is a thin gray matter that is located between the insular cortex and the striatum; it extends in the anterior-posterior and dorsoventral directions (Mathur, 2014). Surprisingly, the claustrum has bidirectional connections with almost all cerebral regions and basolateral amygdala (White et al., 2017). Although the involvement of claustrum in the RHI has not been reported so far, the region has been suggested to be involved in multimodal sensory integration, saliency detection, attentional load allocation, and switch-on/off of consciousness (Calvert, 2001; Crick and Koch, 2005; Mathur, 2014; Dillingham et al., 2017; Smith et al., 2019). These broad connections indicate the possibility that the claustrum might be related to the visuotactile integration for the modulation of body consciousness. However, density of c-Fos positive cells in the claustrum was not significantly different between in the WT mice and *Caps2*-KO mice. This result indicates that this region is not critical for the weakening of RTI response in *Caps2*-KO mice.

In addition, an increase in the densities of c-Fos positive cells were observed in the contralateral primary somatosensory (SI) cortex of the WT mice exposed to the synchronous condition. One possibility is that synchronous presentation of visual and tactile stimuli amplified the response of the SI, because activity of the SI cortex would be modulated by visual inputs via the thalamus or direct connections from the visual cortex (Allen et al., 2017; Masse et al., 2017). Therefore, there is a possibility that grasping of the rubber tail might cause the activation of the SI cortex. As previously mentioned, in monkeys, neurons in the SI and primary motor cortices respond to virtual touches alone after they occurred synchronously with physical brushes to monkeys’ arms (Shokur et al., 2013). However, this was not the case in the present experiment, because c-Fos expression in the tail region of SI of the WT mice exposed to the synchronous condition was not significantly high. Consistently, human neuroimaging studies have not reported activations in the hand region of SI during the RHI (Ehrsson et al., 2004, 2005; Gentile et al., 2013; Guterstam et al., 2019). Thus, another possibility is rather plausible. For example, an increase in spatial attention by the RTI would cause whisking behavior to the side of the rubber tail, because whiskers are important for exploratory behavior (Deschenes et al., 2012). In the present study, an increase in the density of c-Fos positive cells on the SI cortex was widely observed on the barrel field of SI. To clarify the reason of SI activation during the RTI, further observation of the behavior (i.e., observation of whisking behavior during the task) and acquisition of physiological measurements for time course and receptive field of each neuron (i.e., electrophysiological or optical imaging experiments) are needed in future studies.

The present study also suggests higher densities of c-Fos-positive cells in the contralateral hippocampus of the *Caps2*-KO mice. A previous study suggests that the number of GABAergic inhibitory neurons and their synapses are decreased in the hippocampus of the *Caps2*-KO mice, while excitatory neurons in the hippocampus are largely unaffected (Shinoda et al., 2011). This might cause overactivation in the hippocampus in specific situations. In addition, higher densities of c-Fos-positive cells in the contralateral amygdala in the asynchronous condition of the WT mice was observed, compared to that in the synchronous condition. We speculate that unpredictable stimuli compared to the synchronous stroking might cause this activation (Herry et al., 2007). Because a recent study suggests that the amygdala reduces susceptibility to the RHI (Spengler et al., 2019), the activation of amygdala in the asynchronous condition might contribute inhibition of occurrence of the RHI.

We compared the c-Fos expression in both the WT and the *Caps2*-KO mice that experienced the synchronous stroking of rubber and actual tails, since a greater difference in the response rates of the RTI response was observed in this condition. Moreover, this enabled to control the amount of brush stroking during the rubber tail task. Because of the limited number of samples, we used this type of comparison instead of measuring interactions between synchronous and asynchronous conditions among the groups. Thus, the present results could potentially include general differences between *Caps2*-KO and WT mice. To investigate the precise interactions, measuring the interaction using the factorial design is needed in future studies.

In this study, we investigated the c-Fos expression in both WT and the *Caps2*-KO mice during the rubber tail task to elucidate the neural basis of the RTI response and its impairment. After comparing densities of c-Fos positive cells of each section among the groups, we found that the c-Fos expression in the PPC cortex was diminished in the *Caps2*-KO mice exposed to synchronous stroking. The decrease in the c-Fos expression in the PPC may suggest that the synchronized activity from visual and tactile inputs was diminished, and it may be related to impaired multisensory integrations in the *Caps2*-KO mice.

## Data Availability Statement

The raw data supporting the conclusions of this article will be made available by the authors, without undue reservation.

## Ethics Statement

The animal study was reviewed and approved by institutional committee for animal experimentation (Research Institute of National Rehabilitation Center for Persons with Disabilities). Written informed consent was obtained from the owners for the participation of their animals in this study.

## Author Contributions

MW and KK conceived the research. MW and MI conducted the experiments. YSh, YSa, and TF provided the mice. MW and KT analysed the data. MW wrote first draft of the manuscript. All authors read and approved the final manuscript.

## Conflict of Interest

The authors declare that the research was conducted in the absence of any commercial or financial relationships that could be construed as a potential conflict of interest.

## Supplementary Material

Supplementary Figure 1 Correlation mapping between c-Fos-positive cell densities and response rates in the synchronous condition of the rubber tail task. With regards to the WT and *Caps2*-KO mice exposed to the synchronous condition (WT Sync + *Caps2*-KO Sync), the Pearson correlation coefficient between the response rate of the synchronous condition in the rubber tail task and c-Fos-positive cell densities were calculated in each matrix of PCDMs. Scale bar: 1 mm.
