# Supplementary figures and images for: Task-Related c-Fos Expression in the Posterior Parietal Cortex During the “Rubber Tail Task” Is Diminished in Ca2+-Dependent Activator Protein for Secretion 2 (Caps2)-Knockout Mice
Source: Front Behav Neurosci. 2021 Jun 10;15:680206. doi: 10.3389/fnbeh.2021.680206 (PMC8222529; doi:10.3389/fnbeh.2021.680206)

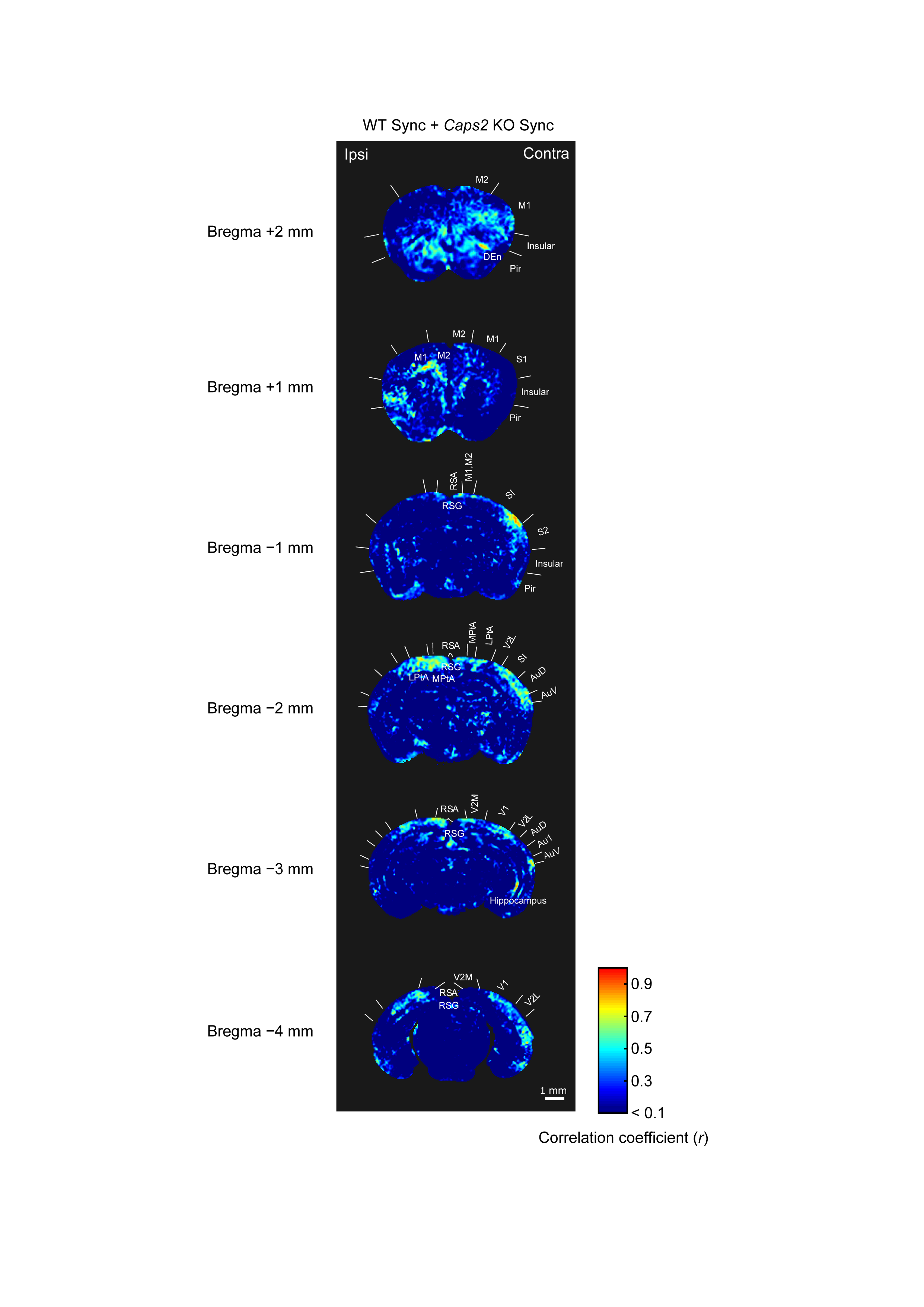

Supplement: Supplementary Figure 1 — Correlation mapping between c-Fos-positive cell densities and response rates in the synchronous condition of the rubber tail task. With regards to the WT and Caps2-KO mice exposed to the synchronous condition (WT Sync + Caps2-KO Sync), the Pearson correlation coefficient between the response rate of the synchronous condition in the rubber tail task and c-Fos-positive cell densities were calculated in each matrix of PCDMs. Scale bar: 1 mm. [file Image_1.TIF]
